# Hepatitis C virus core, NS3, NS4B and NS5A are the major immunogenic proteins in humoral immunity in chronic HCV infection

**DOI:** 10.1186/1743-422X-6-84

**Published:** 2009-06-23

**Authors:** Maarit Sillanpää, Krister Melén, Päivi Porkka, Riku Fagerlund, Kaisu Nevalainen, Maija Lappalainen, Ilkka Julkunen

**Affiliations:** 1 Department of Vaccination and Immune Protection, National Institute for Health and Welfare (THL), P.O. Box 30, FI-00271 Helsinki, Finland; 2 Department of Virology, Laboratory Services (HUSLAB), Helsinki University Hospital, FI-00014 Helsinki, Finland

## Abstract

**Background:**

The viral genome of hepatitis C virus constitutes a 9.6-kb single-stranded positive-sense RNA which encodes altogether 11 viral proteins. In order to study the humoral immune responses against different HCV proteins in patients suffering from chronic HCV infection, we produced three structural (core, E1 and E2) and six nonstructural proteins (NS2, NS3, NS4A, NS4B, NS5A and NS5B) in *Sf*9 insect cells by using the baculovirus expression system.

**Results:**

The recombinant HCV core, E1, E2, NS2, NS3, NS4A, NS4B, NS5A and NS5B proteins were purified and used in Western blot analysis to determine antibody responses against individual HCV protein in 68 HCV RNA and antibody positive human sera that were obtained from patients suffering from genotype 1, 2, 3 or 4 infection. These sera were also analysed with INNO-LIA Score test for HCV antibodies against core, NS3, NS4AB and NS5A, and the results were similar to the ones obtained by Western blot method. Based on our Western blot analyses we found that the major immunogenic HCV antigens were the core, NS4B, NS3 and NS5A proteins which were recognized in 97%, 86%, 68% and 53% of patient sera, respectively. There were no major genotype specific differences in antibody responses to individual HCV proteins. A common feature within the studied sera was that all except two sera recognized the core protein in high titers, whereas none of the sera recognized NS2 protein and only three sera (from genotype 3) recognised NS5B.

**Conclusion:**

The data shows significant variation in the specificity in humoral immunity in chronic HCV patients.

## Background

Hepatitis C virus (HCV) is classified in the *Hepacivirus *genus within the *Flaviviridae *family. The viral genome constitutes a 9.6-kb single-stranded positive-sense RNA with 5' and 3' noncoding regions and a long open reading frame encoding a polyprotein precursor of about 3,000 amino acids in length. The HCV polyprotein precursor is co- and post-translationally processed by cellular and viral proteases to yield 11 viral proteins [1,2]. The structural HCV proteins include the core protein and transmembrane glycoproteins, E1 and E2. The core region also encodes for an alternative open reading frame protein (ARFP) or F protein whose function is presently not known [1,3]. The region between the structural and non-structural genes encodes for an integral membrane cation channel protein p7 [4] which is essential for virus production [5]. HCV has six nonstructural proteins; NS2, NS3, NS4A, NS4B, NS5A and NS5B (see for reviews; [2,6]. NS2 is a cysteine protease responsible for an autoproteolytic NS2–NS3 cleavage and it requires the aminoterminal one-third of NS3 for its enzymatic activity. NS3 is a multifunctional protein with both serine protease and RNA helicase/NTPase activities and NS4A is as an essential cofactor for NS3 protease functions. Currently, there is little information of the function of NS4B protein, but it participates in the formation of a membranous web where HCV RNA replication is suggested take place [6,7]. NS5A is a phosphoprotein which takes part in virus particle formation and is involved in virus resistance against interferons [8]. The NS5B protein encodes for an RNA-dependent RNA polymerase (RdRp), which is the central catalytic enzyme of the HCV replicase [9,10].

Generally, HCV is divided into six major genotypes (or clades) that can be further divided into several subtypes from A to L [11,12]. The amino acid sequences of the major HCV genotypes differ approximately 30% from each other [11]. The geographical distribution of HCV genotypes is also diverse. The genotypes 1, 2 and 3 are found throughout the world whereas the distribution of the other genotypes is much more restricted; genotype 4 is found in the Middle East and Africa, genotype 5 in South Africa and genotype 6 in Southeast Asia [11,13]. In the United States less than 1% of HCV patients are infected with the HCV genotypes 4, 5 or 6 [14]. However, the epidemiology of HCV infection is changing continuously, which is e.g. seen in a manner that the number of genotype 4 infected patients has increased in Europe as a consequence of increasing immigration and intravenous drug use during the last 15 years [15]. The overall worldwide prevalence of HCV is approximately 3%. The highest HCV prevalence figures up to 10–20%, are found in Egypt where the genotype 4 is the most common one [16]. The prevalence of HCV infection varies remarkably and for instance in different European countries it ranges from 0,1% to 4% [15]. Acute HCV infection can be cleared spontaneously only in up to 15–30% of the cases, while usually the infection becomes chronic. Within 20 to 30 years chronic HCV infection can progress to cirrhosis in 20% of the patients leading to hepatocellular carcinoma roughly in yearly rate of 1–4%. Although the commercial methodology to detect HCV-specific RNA and antibody responses in patient sera has greatly advanced in recent years there is no detailed information of the immunogenicity of different HCV proteins in patients suffering from chronic HCV infection.

In the present work, we have described the expression and purification of nine different recombinant HCV proteins in insect cells and analyzed humoral immune response against each viral protein using Western blotting in patients suffering from chronic HCV infection of genotypes 1, 2, 3 or 4. We found that most of the 68 HCV RNA and antibody positive patient sera studied recognized the core, NS3, NS4B and NS5A proteins with high titers. Instead, only three sera recognised NS5B and none of the sera recognized NS2 protein. These results show that antibody responses to various HCV proteins show considerable qualitative and quantitative differences with certain proteins being highly immunogenic in practically all HCV-infected individuals while certain proteins such as NS2 and NS5B were virtually devoid of all immunogenic activity.

## Methods

### Cell culture

Monolayers and suspension cultures of *Spodoptera frugiperda Sf*9 cells were maintained in TNM-FH medium and 10% fetal calf serum (Integro, Zaandam, Netherlands) as described [17].

### Construction of expression plasmids for different HCV genes

Different HCV genes were amplified with PCR from pBRTM/HCV1-3011 [18] carrying the HCV genotype 1b cDNA, and the PCR products were subcloned into the *Bam*HI site of the pcDNA3.1(+)-FLAG plasmid under CMV promoter [19]. The primers (Dako A/S, Glostrup), which were used to modify the 5' and 3'ends of core, E1, E2, NS2, NS3, NS4A, NS4B, NS5A, and NS5B genes have been described elsewhere [20]. After partial sequencing, the HCV protein-coding cDNAs (core, E1, E2, NS2, NS3, NS4A, NS4B, NS5A, NS5B) were subcloned into the *Bam*HI site of the pAcYM1 baculovirus transfer vector under the control of polyhedrin promoter [17]. NS2 protein was expressed with a His-tag. To create recombinant HCV protein-expressing viruses pAcYM1 expression constructs were cotransfected with linearized baculovirus DNA using BaculoGold™ Transfection Kit (PharMingen, San Diego, CA) and recombinant viruses were obtained. All DNA manipulations were performed according to standard protocols.

*In vitro *translation of the HCV genes cloned into pcDNA3.1(+)-FLAG plasmid constructs was carried out with T7 Cap-Scribe and reticulocyte translation kit (Boehringer Mannheim GmbH, Mannheim, Germany). After translation, the samples were diluted in Laemmli sample buffer and analyzed by SDS-PAGE.

### Production and purification of recombinant HCV proteins

*Sf*9 cells were grown to confluence in plastic cell culture bottles (175 cm^2^), infected with HCV core, E1, E2, NS2, NS3, NS4A, NS4B, NS5A, and NS5B expressing recombinant baculoviruses for 1 h and grown for 72 h to produce the recombinant proteins [17]. The cells were collected by centrifugation at 1500 rpm for 10 min followed by washing with phosphate-buffered saline (PBS). The cells were processed further immediately or stored at -70°C. The cells were sonicated on ice, and concentrations of total cellular proteins were quantified with the Bio-Rad protein assay (Bio-Rad Laboratories, Richmond, CA). Expression of recombinant HCV proteins was verified with Coomassie Blue staining, metabolic labeling with [^35^S]-methionine, and Western blotting.

5 mg of sonicated, cellular protein samples in Laemmli sample buffer was purified using preparative SDS-PAGE (Model 491 Prep Cell, Bio-Rad Laboratories). The recombinant HCV core, E1, E2, NS2, NS3, NS4A, NS4B, NS5A, and NS5B proteins were separated on 6 to 15% gradient SDS-PAGE. The sample fractions containing separated proteins from preparative SDS-PAGE were first lyophilized followed by resuspension into 0.5 ml of water. The purity and quantity of each sample fraction were verified with Coomassie Blue staining (compared to known standard protein) and with Western blotting, using specific immunosera. To reduce the amount of SDS in the lyophilized samples, each protein fraction was concentrated with Millipore protein concentration kit UFV5BCC25 (Millipore, Bedford, MA).

### HCV antibodies

Primary antibodies used in Western analysis were rabbit anti-HCV core and NS5A [20], mouse anti-FLAG M5 (for the detection of *in vitro *translated HCV E1, E2, NS2, NS3, NS4A, NS4B, and NS5B proteins; Sigma Chemical Co., St. Louis, MO) and mouse anti-Penta-His (for the detection of 6xHis-NS2; Qiagen, Venlo, Netherlands). Secondary Abs were HRP-conjugated goat anti-rabbit and anti-mouse immunoglobulin (Jackson ImmunoResearch Laboratories, Inc., West Grove, PA) and HRP-conjugated goat anti-human IgG (H+L) (Vector Laboratories, Inc., Burlingame, CA).

### HCV positive and negative human sera

Altogether 68 HCV RNA and antibody positive patients with various HCV genotypes were studied. Five of these patients were treated with interferon-α monotherapy [21,22] for 12 months in the case of genotype 1 infection and for 6 months in genotype 3 infection. The serum samples from these five patients were collected in the beginning and in the end of treatment and also 6 and/or 12 months after treatment. HCV antibodies were determined with commercial tests according to the manufacturer's instructions (Architect Anti-HCV, Abbott, Wiesbaden, Germany; Innotest HCV Ab IV, Innogenetics, Ghent, Belgium; Inno-LIA HCV Ab III update, Innogenetics, Ghent, Belgium). HCV RNA detection was performed by Cobas Amplicor HCV Test, Roche, and genotyping by Inno-LIPA, Innogenetics, Ghent, Belgium. Samples from 50 HCV antibody negative patients served as negative controls.

For safety reasons HCV RNA and antibody positive and HCV antibody negative human sera from patients were inactivated by heating the samples at 56°C for 1 h in the presence of 0.1% Triton X-100. To avoid repeated freezing and thawing an equal volume of 100% glycerol was added on inactivated sera and the 1:2 diluted serum specimens were stored at -20°C in a liquid form.

### Detection of HCV antibodies in human sera by Western blotting using baculovirus-produced recombinant HCV proteins and commercial INNO-LIA Score test

To analyse humoral immune responses against HCV proteins serum specimens were obtained from 68 HCV RNA and antibody positive patients. For Western blot analysis 1 μg of each purified HCV protein was loaded onto two Novex pre-cast, preparative 10–20% Tris-glycine polyacrylamide gels (Invitrogen Corp., Carlsbad, CA). The core (21 kDa), NS2 (24 kDa), NS3 (68 kDa), NS4A (6 kDa), NS4B (29 kDa) and NS5A (49 kDa) proteins were loaded on one gel, and E1 (21 kDa), E2 (40 kDa) and NS5B (64 kDa) proteins to another gel. 10 μg of *Sf*9 cell extract was also loaded onto a separate gel as a control. Proteins separated on gels were transferred onto Immobilon-P membranes (polyvinylidine difluoride; Millipore) with an Isophor electrotransfer device (Hoefer Scientific Instruments, San Francisco, CA). The membranes were sliced and stained with HCV RNA and antibody positive patient sera (dilutions of 1:100, 1:500, 1:2500, 1:12500 and 1:62500) in PBS containing 5% nonfat milk at room temperature for 2 h. After washing with PBS, secondary peroxidase-conjugated anti-human IgG antibodies (Vector Laboratories, Burlingame, Inc., CA) were allowed to bind at room temperature for 1 h. After washing with PBS, the bands were visualized by 3-amino-9-ethylcarbazole (AEC) [23] or the enhanced chemiluminescence system (ECL) (Amersham, Buckinghamshire, UK) as recommended by the manufacturer.

Also 50 HCV negative human sera were analysed as controls. 1 μg of each purified core, NS3, NS4B and NS5A HCV proteins was loaded onto 15% SDS-PAGE gels and blotted to Immobilon-P membranes. The membranes were sliced and stained with negative human sera diluted in 1:100 and 1:500.

These HCV RNA and antibody positive and HCV antibody negative human serum samples were also analysed with INNO-LIA™ * HCV Ab III update or INNO-LIA™ * HCV Score test according to the manufacturer's instructions (Innogenetics, Ghent, Belgium).

## Results

### Production of recombinant HCV proteins in insect cells

**Figure 1:**
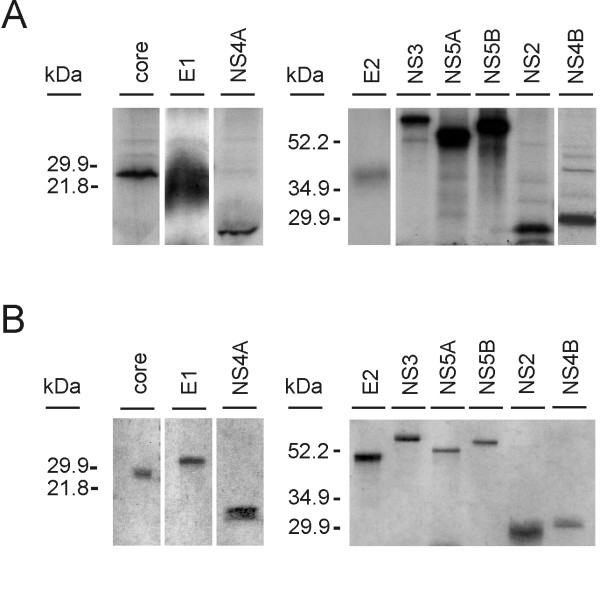
**Expression of recombinant HCV proteins**. A. Individual HCV genes were cloned into pcDNA3.1(+) plasmid under CMV promoter. The expression of HCV proteins was verified by in vitro translation. The proteins were metabolically labeled with [^35^S]-methionine and separated on 15% (core, E1, NS4A) or on 12% (E2, NS2, NS3, NS5A, NS5B, NS4B) SDS-PAGE and autoradiographed. B. SDS-PAGE analysis of purified recombinant HCV proteins. Individual HCV genes were inserted into baculovirus expression plasmids and recombinant HCV-expressing baculoviruses were obtained. Recombinant HCV proteins were produced in *Sf9 *cells followed by purification of the proteins by preparative SDS-PAGE. Samples of purified HCV proteins (0.5–1 μg each) were separated on 15% (core, E1 and NS4A) or 12% (E2, NS3, NS5A, NS5B, NS2 and NS4B) SDS-PAGE and stained by Coomassie Blue.

To produce recombinant HCV proteins individual HCV genes from genotype 1b cDNA were amplified with PCR and the products were subcloned into the pcDNA3.1(+)-FLAG plasmid, followed by in vitro translation and verification of the translation products by SDS-PAGE and autoradiography (Fig. 1A). Next the PCR fragments encoding for different HCV proteins were subcloned into the pAcYM1 baculovirus transfer vector, and baculoviruses expressing the recombinant core, E1, E2, NS2, NS3, NS4A, NS4B, NS5A, and NS5B proteins were constructed. *Sf9 *cells were infected with recombinant baculoviruses and different HCV proteins were purified with preparative SDS-PAGE. The purified recombinant HCV proteins are shown on Coomassie Blue-stained polyacrylamide gels (Fig. 1B).

### Recombinant HCV proteins in the analysis of HCV-specific humoral immune responses in human sera

**Figure 2:**
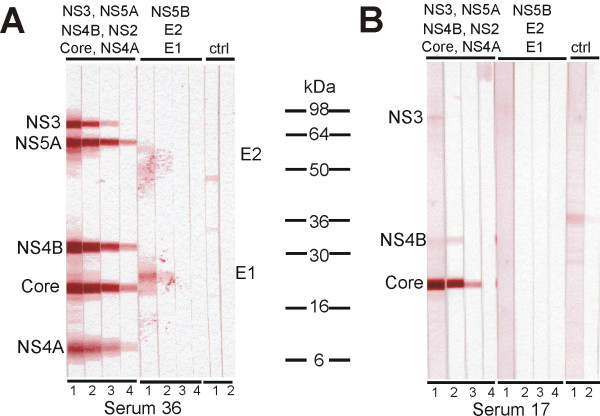
**Detection of anti-HCV antibodies in HCV RNA and antibody positive human sera with recombinant HCV proteins**. 1 μg of each baculovirus-expressed and preparative SDS-PAGE-purified recombinant HCV protein was loaded onto 10–20% Tris-glycine polyacrylamide gradient gels. Core, NS2, NS3, NS4A, NS4B, and NS5A were loaded on one gel, and E1, E2, and NS5B on another gel, respectively. 3 μg of *Sf*9 cell extract was also loaded onto one gel as a control. Proteins separated on gels were transferred to nylon membranes, sliced and stained with serially diluted human serum obtained from HCV RNA and antibody positive patients. The following dilutions were used (lane 1) 1:100, (lane 2) 1:500, (lane 3) 1:2500, (lane 4) 1:12500 and 1:62500 (not shown). After incubation with secondary Abs, the bands were visualized by 3-amino-9-ethylcarbazole (AEC). A. An example of highly positive human serum number 36 is shown, B. an example of a weakly positive human serum number 17 is shown.

The availability of recombinant baculovirus-produced HCV proteins enabled us to analyze antibody responses against nine different HCV proteins in HCV-positive individuals by Western blotting. The purified recombinant HCV proteins were loaded into two gels for analysis; core, NS2, NS3, NS4A, NS4B and NS5A in one and NS5B, E1 and E2 in another gel (see Fig. 2.). The proteins were transferred onto nylon membranes, which were sliced and used for the analysis of HCV-specific antibody responses in different serum dilutions (from 1:100 to 1:62500). As an example of individual differences in the quality and quantity of anti-HCV antibodies serum specimens showing antibodies against multiple HCV proteins (serum 36; Fig. 2A), or only few of them (serum 17; Fig. 2B) are shown. For comparison the patient serum samples were also analysed with a third generation immunoassay INNO-LIA HCV Score test which contains HCV antigens for the core, E2, NS3 and NS5A as well as a combination of NS4A and NS4B. The comparison of the results of our Western blot analysis and INNO-LIA Score test is shown in Table 1. In general, both tests recognized core, NS4 and NS5A-specific antibodies in the same samples with only few exceptions. The INNO-LIA HCV Score test appeared to be somewhat more sensitive in the case of NS3 and E2 proteins since 21 serum samples more were found to be positive with this method as compared to Western blot analysis (Table 1).

**Table 1:** Correlation of anti-HCV antibody patterns by Western blot analysis and INNO-LIA Score test.

	Western blot analysis		INNO-LIA Score test		Correlation of tests*	
			
	+	-	+	-	+/+	-/-
			
Core	66	2	66	2	65	1
E2	21	47	37	31	16	26
NS3	47	21	68	0	47	0
NS4A+B	61	7	63	5	60	4
NS5A	36	32	31	37	27	28

The prevalence of anti-HCV antibodies in the 68 HCV RNA and antibody positive human sera were analysed by Western blot analysis with recombinant HCV proteins and by commercial INNO-LIA Score test.

* The number of serum specimens giving a positive or a negative result with both tests is shown with +/+ or -/-, respectively.

Also 50 HCV antibody negative human sera were diluted in 1:100 and 1:500 and analysed with the Western blot method using recombinant HCV core, NS3, NS4B or NS5A proteins, which represent the major immunogenic proteins of HCV (see below). There was some faint staining in 11 HCV-negative samples against certain individual HCV proteins, while no antisera recognized the core protein, which represents the major HCV immunogenic protein (see below). When these samples were analysed with INNO-LIA HCV Score test the results were considered negative.

### The core, NS3, NS4B and NS5A proteins form the major immunogenic proteins of HCV virus

**Figure 3:**
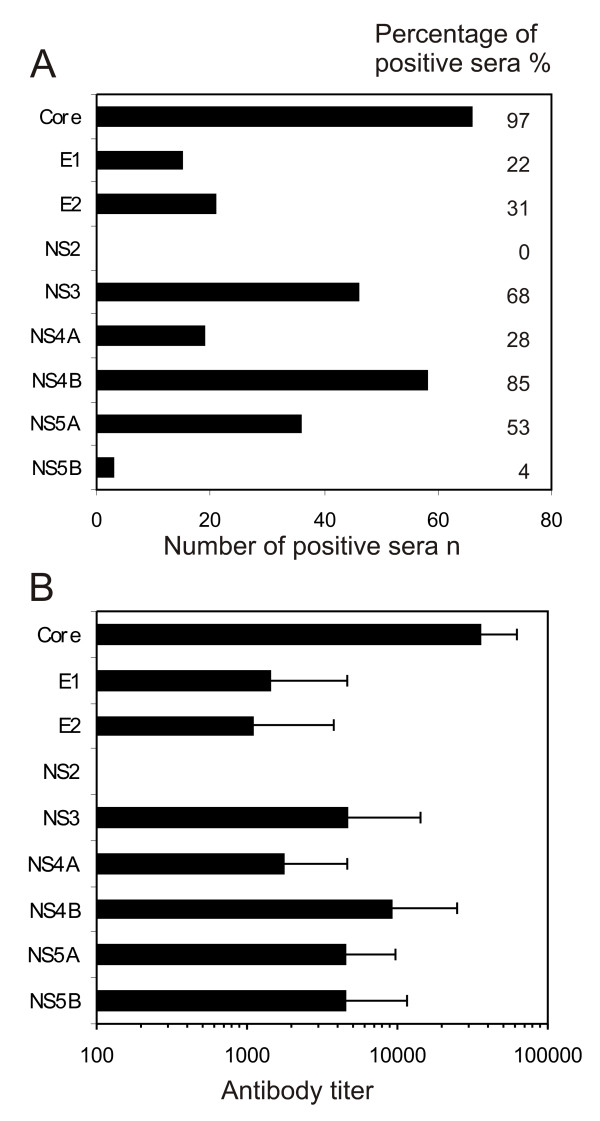
**The specificity of anti-HCV antibody responses in patients suffering from chronic HCV infection**. A. The frequency of antibodies against individual recombinant HCV proteins in 68 serum specimens obtained from patients suffering from chronic HCV infection. Both the number and percentage of positive sera are shown. B. The relative antibody levels against individual HCV proteins were determined as the last serum dilution showing a positive signal in Western blot analysis. The means and standard deviations of the means for antibody levels against individual HCV proteins are shown based on 68 HCV RNA and antibody positive patient sera. Only individuals showing a positive antibody response against a given HCV protein are included into the means.

The frequency of antibody responses against individual HCV proteins is shown in Fig. 3A. From the 68 HCV RNA and antibody positive patient sera studied, 97% recognized the core, 85% NS4B, 68% NS3 and 53% NS5A proteins (Fig. 3A). When the antibody levels were determined as the last serum dilution giving a positive staining in Western blot analysis, the highest mean antibody titer of approximately 1:50 000 (+/- 15 000) were found against the core protein, while the mean antibody titers against NS3, NS4B, and NS5A proteins were on an average 10-fold lower (Fig. 3B). The next common immunogenic proteins were E2, NS4A and E1 which were recognized by 31%, 28% and 22% of the sera, respectively with mean antibody titers ranging between 1:1000 to 1:2500 (Fig. 3). The remaining HCV proteins were very poorly immunogenic and only three serum specimens recognized NS5B protein with a mean antibody titer of 1:5000 and none of the sera recognized NS2 protein.

### Genotype-specific differences in anti-HCV antibody responses

Next the HCV RNA and antibody positive serum samples were grouped according to the HCV genotypes; 21 sera of genotype 1, 20 sera of genotype 2, 23 sera of genotype 3 and 4 sera of genotype 4. When antibody responses against individual HCV proteins were classified in a genotype-specific manner, some variation in genotype-specific responses was seen (Table 2). There were only four patients infected with HCV genotype 4 and therefore the information on genotype 4 may be considered suggestive. The sera from genotype 1 infected patients recognized more often E1, E2 and NS3 proteins than the serum specimens from other genotypes. This is logical since recombinant HCV proteins were of genotype 1 origin. When genotypes 2 and 3 were compared, antibodies against recombinant core, NS4A and NS5A were found as often in both genotypes. However, antibodies against E1 and E2 proteins were found more often in genotype 2 samples and antibodies against NS3, NS4B and NS5B proteins were found more often in genotype 3 samples (Table 2). Antibodies for recombinant NS5B protein were found only in three serum samples that represented HCV genotype 3. As a whole there was some variation in the ability of different HCV genotypes to recognize recombinant HCV proteins.

**Table 2:** Hepatitis C virus genotype-specific antibody responses against nine recombinant HCV proteins.

		**Percentage of positive sera of each genotype**								
		
**Genotype**	n	Core	E1	E2	NS2	NS3	NS4A	NS4B	NS5A	NS5B
**1**	21	95	43	52	0	90	52	95	57	0
**2**	20	95	20	25	0	50	15	70	50	0
**3**	23	100	4	13	0	67	13	87	52	13
**4**	4	100	25	50	0	50	25	100	25	0

The prevalence of anti-HCV antibodies were analysed by Western blot analysis with recombinant HCV proteins in 68 patients with chronic HCV infection caused by different genotypes 1, 2, 3 and 4. The data is presented as the percentage of tested sera in each genotype that showed antibody response against a given HCV protein. The number of analysed sera for each genotype is shown as n.

**Figure 4:**
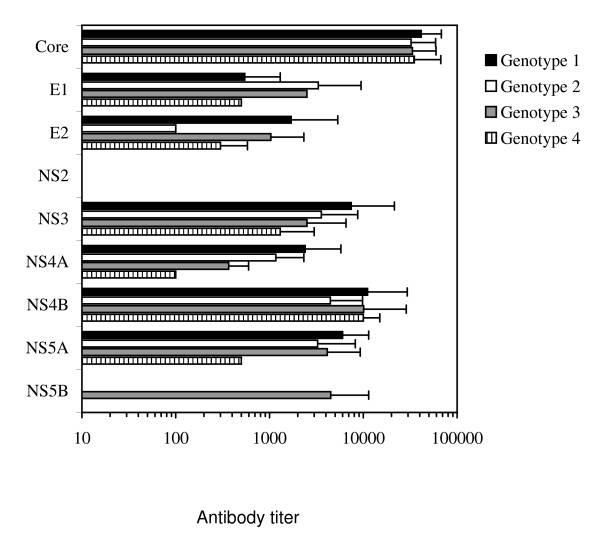
**Anti-HCV antibody responses in patients infected with different HCV genotypes**. The mean antibody titers and standard deviations against individual recombinant HCV proteins when samples were grouped according to the HCV genotypes 1, 2, 3 or 4. The analysis contained 21 sera of genotype 1, 20 sera of genotype 2, 23 sera of genotype 3 and 4 sera of genotype 4. Only those serum specimens showing a positive response to a given HCV protein are included in the means.

Also the mean antibody titers against individual recombinant HCV proteins were calculated for different HCV genotypes (Fig. 4). In the case of core and NS4B proteins the antibody titers against these proteins were practically even between the different genotypes. There was some variation when the antibody titers of E1, E2, NS3, NS4A and NS5A were compared between different HCV genotypes. Certain differences were seen in genotype 4 that was represented only by four samples and therefore it is difficult to estimate the reliability of these differences. It was also of interest that serum specimens obtained from patients suffering from genotype 2 HCV infection had lower antibody titers against recombinant E2 protein as compared to serum specimens from patients suffering other genotype infections.

### Individual anti-HCV antibody patterns remain relatively constant during the follow-up

**Figure 5:**
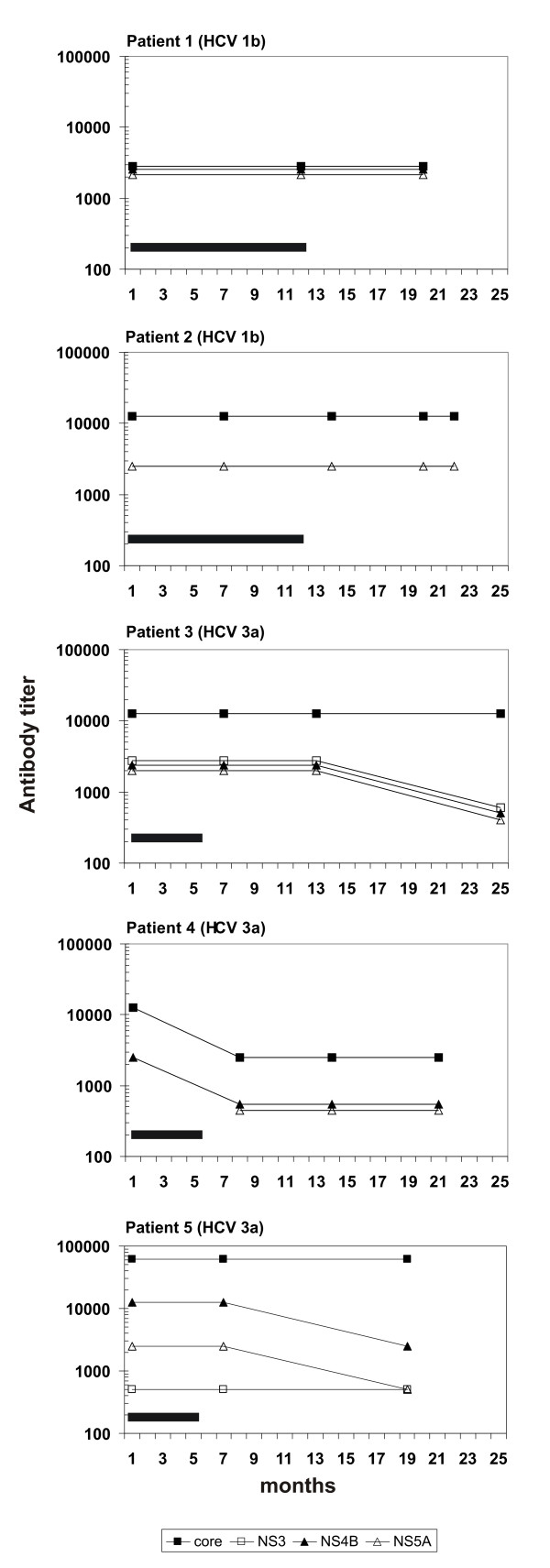
**The persistence of anti-HCV antibody responses against individual HCV proteins**. HCV antibody prevalence in five HCV RNA and antibody positive individuals infected with genotype 1b or 3a was analyzed from serum specimens obtained before and after IFN-α monotherapy (black bar). Relative anti-HCV antibody titers were determined by Western blot analysis as described in Fig. 2.

It is possible that the quality and quantity of anti-HCV antibodies change during the course of natural HCV infection. To consider this possibility we determined anti-HCV antibody levels against all nine HCV proteins in serial serum specimens obtained from five individuals suffering from a chronic HCV infection. Specimens from three individuals with HCV genotype 3a and two individuals with genotype 1b infection were followed-up during and after IFN-α monotherapy. In genotype 1 infected patients the quality and quantity of anti-HCV antibodies remained fully stable, while in genotype 3 infected individuals the antibody levels had a weak tendency to decrease after IFN-α monotherapy (Fig. 5). None of the IFN-α treated patients turned HCV RNA negative.

## Discussion

In the present work, we have expressed recombinant HCV core, E1, E2, NS2, NS3, NS4A, NS4B, NS5A and NS5B proteins by baculovirus system in insect cells. This expression system was chosen in order for the recombinant proteins to undergo all possible posttranslational modifications such as glycosylation and phosphorylation. In addition, in contrast to proteins expressed in *E. coli*, humans are likely to have very low or nonexisting antibodies against insect cell proteins that might be contaminating the recombinant protein preparations. The expression levels of individual HCV proteins were relatively high and they could be purified by preparative SDS-PAGE (Fig. 1). Some, but not all HCV genomes are also encoding protein F from an alternative reading frame of the core sequence. For unknown reasons we were not able to produce the F protein by baculovirus expression and therefore we could not include this protein in our analyses. We also did not express the small ion channel protein p7.

We used full-length recombinant HCV proteins from HCV genotype 1b to analyze antibody responses against individual viral proteins in patients suffering from chronic infection caused by HCV genotypes 1, 2, 3 or 4. Our analyzes revealed that all recombinant HCV proteins except that of NS2 were immunogenic in humans and there were no major differences in the magnitude of immune responses at least against the core and NS proteins between the different genotype infections. It was of interest that NS2 protein appears to completely lack immunogenicity in humans. This was unexpected, but yet we are confident with the results, since the sequence of NS2 expression construct was correct and monoclonal anti-NS2 antibodies readily detected the transiently expressed protein [24]. This may indicate that in humans there may be proteases or other molecules homologous to NS2 leading to an inability of the host to recognize the NS2 protein as foreign. Further evidence that baculovirus expressed recombinant proteins of HCV genotype 1b are suitable for immunological analyses was obtained from the comparison of our Western blot analysis with the commercial INNO-LIA Score test, which is able to detect antibodies from genotypes 1–5. These methods showed a very good correlation in the case of anti-core, NS4A+B and NS5A antibody responses. This suggests that the baculovirus produced HCV proteins provide valuable and very specific research reagents for analyzing HCV-specific immune responses against HCV. However, the INNO-LIA Score test was more sensitive than the Western blot method in the case of anti-NS3 and E2 antibodies. The reason for this discrepancy is not known, but it may be that the relative amount of HCV antigens used in the INNO-LIA assay was higher that what we used in the Western blot analysis. In addition, the conformation of the recombinant proteins may also contribute to the results, since it is known that many antigenic epitopes in viral envelope glycoproteins like the E2 of HCV are likely conformational and thus these sites are not necessarily detected by antibodies in denatured proteins. By increasing the amount of viral antigens in Western blot analysis we could theoretically have been able to enhance the sensitivity of our analysis. However, the idea in our analysis was to systematically study the immunogenicity of various HCV proteins in order to reveal which viral proteins are the targets of humoral anti-HCV immune responses in humans. In order to detect the relative immunogenicity of different HCV proteins we used a similar amount of each purified protein in the assay. Based on this analysis we were able to determine qualitative and quantitative differences in host antibody responses to different HCV proteins, which had not been systematically studied before.

Previously, anti-HCV antibody responses have been analysed in acute and chronic phases of HCV infection [25-28]. In the present study we focused on patients suffering from a chronic HCV infection and we found remarkable differences in the frequency of anti-HCV antibody responses as well as there was a lot of variation in antibody titers against individual HCV proteins (Fig. 3). We found out that 97% of the sera studied recognized the core protein in very high levels, whereas the other proteins such as the NS4B, NS3, NS5A and E2 were found to be immunogenic in 85% to 31% of the cases, respectively (Fig 3A). A study carried out by Chen and coworkers among 60 chronic HCV patients, revealed E2 antibodies in 98%, core in 97%, NS3 in 88%, NS5 in 68% and NS4 in 48% of the cases [27]. As analyzed by EIA the highest antibody levels were observed against the core protein (ca. 1:5000), while the antibody responses against other viral proteins or peptides derived of them remained at a lower level [27]. As a whole the results of the above study are concordant with the observations of the present study, except that our Western blot analysis gave up to 10-fold higher titers against the core proteins and several fold higher levels of specific antibody responses against other HCV proteins. Also Nikolaeva and coworkers observed the core protein to be highly immunogenic (antibody titers up to 1:40 000) while other HCV proteins were less important immunogens in chronic HCV patients [25]. Direct comparisons of the frequencies and antibody levels to individual HCV proteins in different studies is very difficult, since the methods to produce and purify viral antigens vary and also the form of the assay to detect anti-HCV antibodies varies from one study to another. In our analysis we decided to use the full-length baculovirus-expressed HCV proteins and Western blot analysis in order to be sure of the specificity of the antibody responses to a given protein. One of the drawbacks of the assay is, however, that only antibodies against linear antigenic epitopes within the denatured proteins are being detected in Western blotting.

The prevalence of anti-HCV antibodies have been followed during the chronic phase of infection [25-27]. When we analysed sera from five HCV RNA and antibody positive patients during a period of 18 to 25 month, the antibody levels against the major immunogenic proteins were found to remain relatively constant. However, in three patients there were some changes in anti-HCV antibody levels, namely a weak decrease in the core and NS-specific antibody levels during the follow-up was seen. Similar analysis by others [27,29] revealed very similar results with highly persistent antibody patterns. While in most cases anti-HCV antibodies remain at a constant level, there were some individuals whose antibody levels showed some fluctuation [27].

## Conclusion

We were able to produce nine structural and non-structural HCV proteins in high levels in *Sf*9 insect cells. These purified recombinant HCV proteins were found to be suitable for analyzing the prevalence of antibodies against individual HCV proteins in human sera obtained from patients suffering from chronic HCV infection. Clearly the core, NS3, NS4B and NS5A represented the major antigenic proteins. By Western blotting antibody responses against the viral glycoproteins, E1 and E2 and the NS4A protein were found less frequently. Curiously, the recombinant NS5B protein was recognized only by three patient sera all of which were from patients infected with HCV genotype 3. It was of interest that NS2 protein, a viral cysteine protease was unable to mount humoral immune responses in our patients. These recombinant HCV proteins will also enable the analysis of cell-mediated immune responses in HCV infection as well as to study whether changes in anti-HCV antibody patterns have a prognostic value in patients suffering from chronic HCV infection.

## Competing interests

The authors declare that they have no competing interests.

## Authors' contributions

MS carried out some of the experiments and drafted the manuscript. KM participated in the design of the study and analysed the results. PP, RF, KN and KM constructed the expression vectors and produced and purified the recombinant HCV proteins and used these proteins to screen the patient sera for HCV antibodies. ML genotyped the HCV positive patient sera and provided the specimens for the study as well as participated in the design of the study. IJ initiated the study, participated in its design and coordination and helped to draft the manuscript. All authors have read and approved the final version of the manuscript.

## References

[B1] Walewski JL, Keller TR, Stump DD, Branch AD (2001). Evidence for a new hepatitis C virus antigen encoded in an overlapping reading frame. RNA.

[B2] Lindenbach BD, Rice CM (2005). Unravelling hepatitis C virus replication from genome to function. Nature.

[B3] Xu Z, Choi J, Yen TS, Lu W, Strohecker A, Govindarajan S, Chien D, Selby MJ, Ou J (2001). Synthesis of a novel hepatitis C virus protein by ribosomal frameshift. EMBO J.

[B4] Griffin SD, Beales LP, Clarke DS, Worsfold O, Evans SD, Jaeger J, Harris MP, Rowlands DJ (2003). The p7 protein of hepatitis C virus forms an ion channel that is blocked by the antiviral drug, Amantadine. FEBS Lett.

[B5] Jones CT, Murray CL, Eastman DK, Tassello J, Rice CM (2007). Hepatitis C virus p7 and NS2 proteins are essential for production of infectious virus. J Virol.

[B6] Moradpour D, Penin F, Rice CM (2007). Replication of hepatitis C virus. Nat Rev Microbiol.

[B7] Gosert R, Egger D, Lohmann V, Bartenschlager R, Blum HE, Bienz K, Moradpour D (2003). Identification of the hepatitis C virus RNA replication complex in Huh-7 cells harboring subgenomic replicons. J Virol.

[B8] Tellinghuisen TL, Foss KL, Treadaway J (2008). Regulation of hepatitis C virion production via phosphorylation of the NS5A protein. PLoS Pathog.

[B9] Bartenschlager R, Lohmann V (2000). Replication of hepatitis C virus. J Gen Virol.

[B10] Lohmann V, Roos A, Korner F, Koch JO, Bartenschlager R (2000). Biochemical and structural analysis of the NS5B RNA-dependent RNA polymerase of the hepatitis C virus. J Viral Hepat.

[B11] Simmonds P (2001). The origin and evolution of hepatitis viruses in humans. J Gen Virol.

[B12] Simmonds P, Bukh J, Combet C, Deleage G, Enomoto N, Feinstone S, Halfon P, Inchauspe G, Kuiken C, Maertens G (2005). Consensus proposals for a unified system of nomenclature of hepatitis C virus genotypes. Hepatology.

[B13] Nguyen MH, Keeffe EB (2005). Prevalence and treatment of hepatitis C virus genotypes 4, 5, and 6. Clin Gastroenterol Hepatol.

[B14] Rustgi VK (2007). The epidemiology of hepatitis C infection in the United States. J Gastroenterol.

[B15] Esteban JI, Sauleda S, Quer J (2008). The changing epidemiology of hepatitis C virus infection in Europe. J Hepatol.

[B16] Kamal SM (2007). Improving outcome in patients with hepatitis C virus genotype 4. Am J Gastroenterol.

[B17] Summers MD, Smith GE (1986). A manual of methods for baculovirus vectors and insect cell culture procedures. Texas Agricultural Experiment Station Bulletin.

[B18] Grakoui A, Wychowski C, Lin C, Feinstone SM, Rice CM (1993). Expression and identification of hepatitis C virus polyprotein cleavage products. J Virol.

[B19] Melen K, Kinnunen L, Julkunen I (2001). Arginine/lysine-rich structural element is involved in interferon-induced nuclear import of STATs. J Biol Chem.

[B20] Melen K, Fagerlund R, Nyqvist M, Keskinen P, Julkunen I (2004). Expression of hepatitis C virus core protein inhibits interferon-induced nuclear import of STATs. J Med Virol.

[B21] Ebeling F, Lappalainen M, Vuoristo M, Nuutinen H, Leino R, Karvonen AL, Lehtola J, Julkunen R, Pohjanpelto P, Farkkila M (2001). Factors predicting interferon treatment response in patients with chronic hepatitis c: late viral clearance does not preclude a sustained response. Am J Gastroenterol.

[B22] Ebeling F, Lappalainen M, Vuoristo M, Nuutinen H, Leino R, Karvonen AL, Lehtola J, Julkunen R, Pohjanpelto P, Tolo H, Farkkila M (2000). Leukocyte interferon-alpha in the treatment of chronic hepatitis C in Finland. Scand J Gastroenterol.

[B23] Harlow E, Lane D (1988). Antibodies: a laboratory manual.

[B24] Kaukinen P, Sillanpaa M, Kotenko S, Lin R, Hiscott J, Melen K, Julkunen I (2006). Hepatitis C virus NS2 and NS3/4A proteins are potent inhibitors of host cell cytokine/chemokine gene expression. Virol J.

[B25] Nikolaeva LI, Blokhina NP, Tsurikova NN, Voronkova NV, Miminoshvili MI, Braginsky DM, Yastrebova ON, Booynitskaya OB, Isaeva OV, Michailov MI, Archakov AI (2002). Virus-specific antibody titres in different phases of hepatitis C virus infection. J Viral Hepat.

[B26] Beld M, Penning M, van Putten M, Lukashov V, Hoek A van den, McMorrow M, Goudsmit J (1999). Quantitative antibody responses to structural (Core) and nonstructural (NS3, NS4, and NS5) hepatitis C virus proteins among seroconverting injecting drug users: impact of epitope variation and relationship to detection of HCV RNA in blood. Hepatology.

[B27] Chen M, Sallberg M, Sonnerborg A, Weiland O, Mattsson L, Jin L, Birkett A, Peterson D, Milich DR (1999). Limited humoral immunity in hepatitis C virus infection. Gastroenterology.

[B28] Netski DM, Mosbruger T, Depla E, Maertens G, Ray SC, Hamilton RG, Roundtree S, Thomas DL, McKeating J, Cox A (2005). Humoral immune response in acute hepatitis C virus infection. Clin Infect Dis.

[B29] Muerhoff AS, Gutierrez R, Kyrk C, Leary T, Schlauder G, Dawson G, Desai SM (2008). Genotype dependence of peptide-based immunoassays for the detection of HCV core antibodies. J Med Virol.

